# Transgluteal approach for excision of pelvic lipoma causing sciatic pain

**DOI:** 10.1093/jscr/rjae704

**Published:** 2024-11-18

**Authors:** Nia Gecheva, Petar Ilkov, Konstantin Uzunov

**Affiliations:** Medical University Sofia, Department of Orthopedics and Trumatology, University Hospital of Orthopedics “Prof. B. Boychev”, 1, St. Georgi Sofiiski, Sofia 1431, Bulgaria; Department of Neurosurgery, University Multiprofile Hospital for Active Treatment and Emergency Medicine “N. I. Pirogov”, Sofia 1606, Bulgaria; Medical University Sofia, Department of Spinal Surgery, University Hospital of Orthopedics “Prof. B. Boychev”, Bul. Nikola Petkov 56, Sofia 1614, Bulgaria

**Keywords:** sciatic nerve, compression, lipoma, transgluteal approach

## Abstract

Sciatic nerve pain, affecting 12%-27% of the general population, often arises from a myriad of etiologies due to the complex anatomy of the sciatic region. The intricate relationship between the sciatic nerve and surrounding structures in the pelvis poses significant challenges in both diagnosis and surgical management. We report two cases of adult female patients presenting with chronic sciatic pain, refractory to conservative treatment. Imaging studies, including magnetic resonance imaging and computed tomography, identified retroperitoneal lipomas exerting compressive effects on the sciatic nerve. Both patients underwent surgical excision of the lipomas via a transgluteal approach with complete resection of the benign tumors in both cases. The successful outcomes in these cases underscore the potential of the transgluteal approach as a novel and effective treatment strategy in the management of complex retroperitoneal tumors causing sciatic pain.

## Introduction

The prevalence of sciatic nerve pain has been reported in 12%-27% in general population, suggesting that many different mechanisms may contribute to the development of sciatic pain. The patient may have variable presentation such as a palpable lump in the gluteal region, intestinal obstruction, and increased frequency of defecation or urination or due to pressure symptoms on nerves and vessels in pelvis causing sciatica or limb edema respectively. The clinical examination may not be helpful in making the diagnosis due to the deep location of this pathology and imaging plays a pivotal role [1].

Lipomas are the most common benign soft tissue tumors, affecting ~2% of the population and constituting 50% of all musculoskeletal soft tissue tumors [2]. These tumors can be classified as either superficial or deep, with superficial lipomas being more common. Deep lipomas tend to be larger and can deform surrounding tissues, while superficial lipomas are typically more circumscribed [3, 4]. Complete resection of lipomas results in a 1% recurrence rate, whereas intramuscular lipomas have a higher recurrence rate of 19%.

The established surgical approaches to the gluteal region include the infragluteal and transgluteal techniques. The transgluteal approach was initially described in the early 20th century with the infragluteal approach subsequently introduced by Stookey [5]. Henry later expanded on the infragluteal technique, cautioning against the transgluteal approach due to concerns over potential disruption of vascular planes [6]. Additionally, there is a risk of damaging branches of the inferior gluteal artery, which could lead to postoperative complications such as gluteal muscle atrophy or dimpling. The surgical approach must be carefully tailored to the lesion's specific anatomical location and size. The primary objective is to maximize exposure while ensuring safe resection margins. Surgical techniques should be selected based on the lesion’s position and the surgeon's experience, ensuring oncologic safety is maintained post-resection [7].

In this case report, we detail two instances of lipomas originating from retroperitoneal adipose tissue that presented with symptoms resembling radiculopathy.

## Case report

We present two cases of females consequently 57 and 55 years old. Both of them had severe pain and paresthesia detected within the L4 to S3 dermatomes, along the long axis of the right leg. Complaints were described as insidious for more than 6 months with no history of trauma. No history of cancer or other diseases representative for the region was presented.

Clinically, Lasègue’s sign was negative bilaterally, and there was no limitation in the range of motion of the hip joint. Physical examination revealed a normal body mass index without any palpable masses or signs of peritonitis. Nevertheless, the patient experienced pain that intensified with straightening of the right leg. Pain radiating down the back of the thigh, exacerbated by dorsiflexion, was also reported, suggesting potential sciatic nerve compression. Hematological and biochemical workups, including complete blood counts, renal function tests, liver function tests, and coagulation profiles, were all within normal limits.

**Figure 1:**
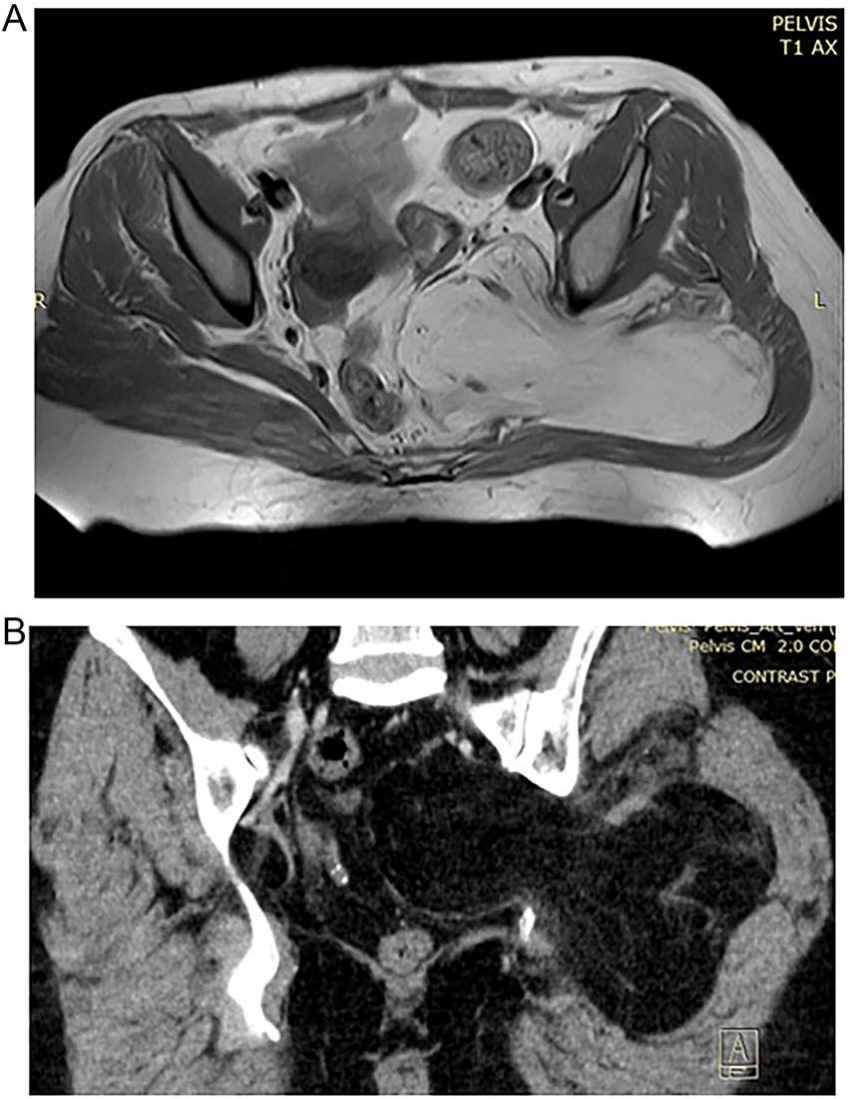
(A) Preoperative MRT image showing lipoma in the pelvic region. (B) Preoperative CT of the 57 year old patient with high signal of the lipomatous lesion.

From the radiological evaluation methods, a computed tomography (CT) scan and magnetic resonance imaging (MRI) were performed for both of the patients;

In the 57-year-old female, CT scan revealed a mass inside the pelvis that invades the gluteal musculature (Fig. 1A and B). The mass was homogeneously isointense with fat and ~155×99×148 in size. MRI revealed space-occupying lesions in the right buttock and pelvic cavity, with clear boundaries and smooth edges, not resulting in the displacement of the pelvic organal components.

**Figure 2:**
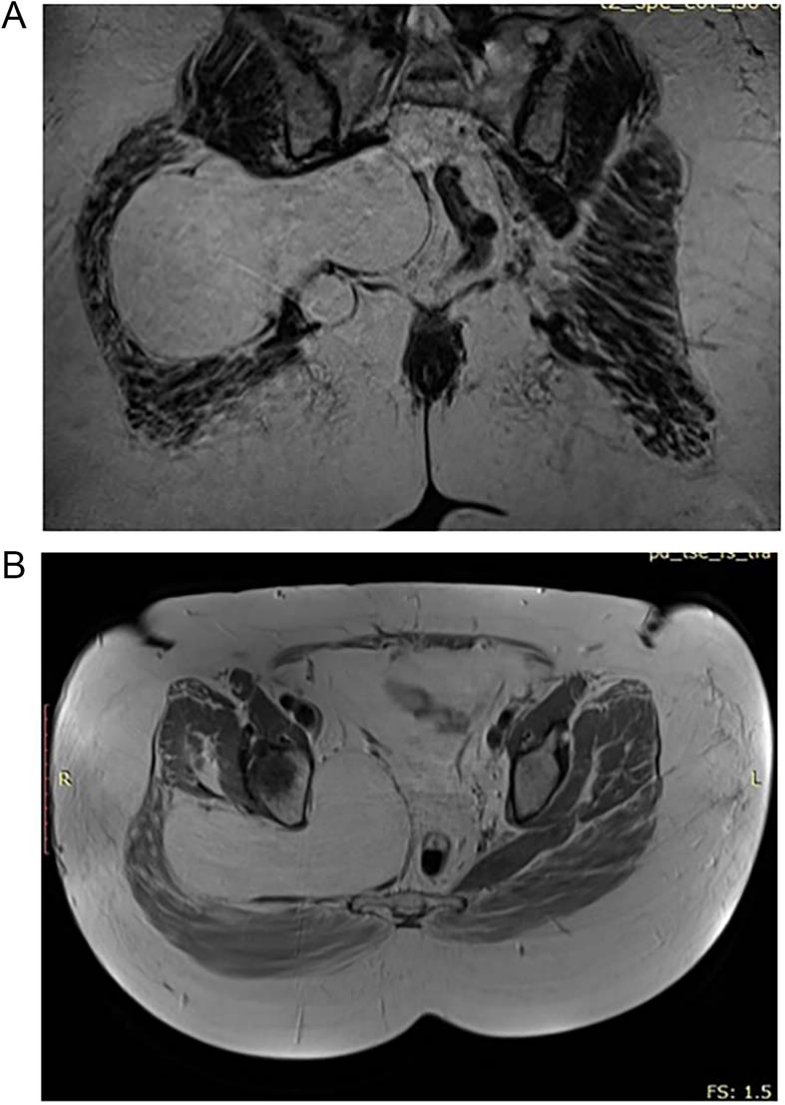
(A) Preoperative CT of the 55 year old patient with lipoma in the pelvic region. (B) Preoperative CT of the 55 year old patient with lipoma in the pelvic region.

In the 55-year-old female, consequently were done a CT scan and MRI imaging (Fig. 2A and B). The patient had a different surgical history compared to the other female representative. She was operated in another medical unit due to spinal stenosis and nerve root decompression following spinal stabilization was performed. However, the complaints were persistent and on a second MRI, the team of neurosurgeons, who performed the operations on both of the presented cases, noticed a mass corresponding with the characteristics of lipoma and being the predominant reason for the complaints as it caused sciatic pain.

Both patients were operated and the masses were eliminated. The complaints resolved within a week of the surgical procedure.

## Approach

**Figure 3:**
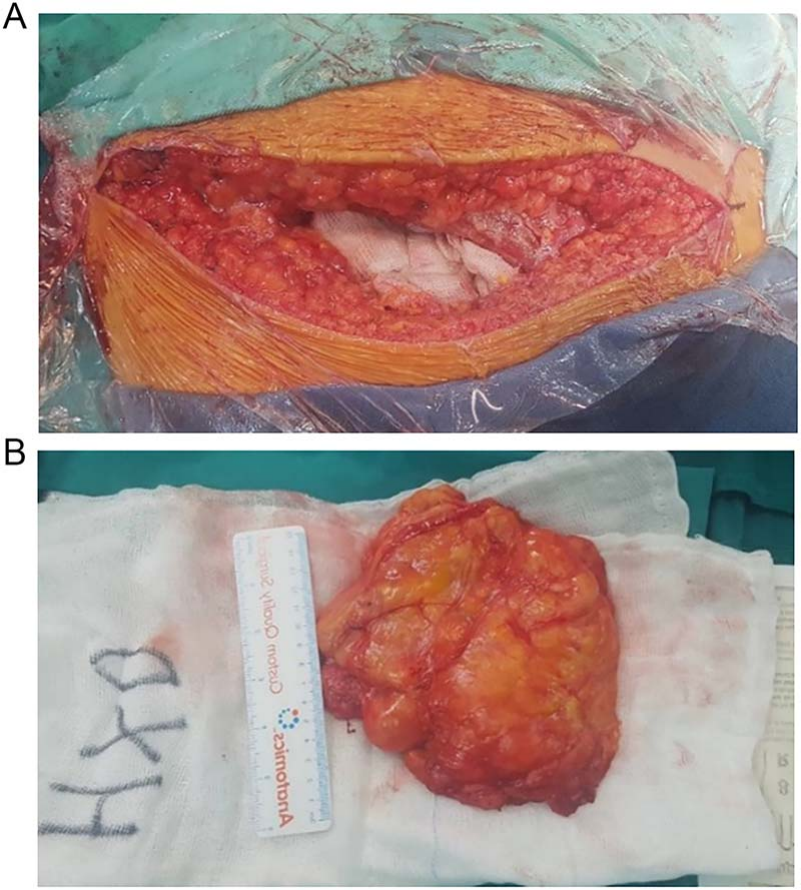
(A) Transgluteal approach used for excision of the lipomatosus lesion. (B) Complete resection of the lesions and intraoperative measurement of its sizes.

A curvilinear incision is made in the direction of the gluteal fibers, centered on the intertrochanteric crest, and extended inferiorly along the femoral shaft. The incision originates 5 cm below and just lateral to the posterior inferior iliac spine, in alignment with the inferior border of the piriformis muscle (Fig. 3A).

Following the initial incision, the gluteal fascia and iliotibial tract are meticulously incised in alignment with the overlying skin incision. Subsequently, the fibers of the gluteus maximus are methodically separated, commencing laterally and advancing medially. Blunt dissection and careful tissue separation enabled effective hemostasis, ensuring a clean and dry surgical field [8].

This technique facilitated the en bloc removal of the capsule along an extracapsular plane, a critical step in achieving the complete resection of the lesion (Fig. 3B).

Wound closure is performed with the gluteal fascia and iliotibial band reapproximated using interrupted sutures to mitigate the risk of muscle herniation. The skin and subcutaneous layers are subsequently closed in a multilayered fashion [9].

Histopathological evaluations confirmed a benign lipoma with localized high fibrous tissue and fat necrosis. No signs of malignancy were identified.

## Discussion

Giant pelvic lipomas in adults are exceedingly rare, with only 17 cases documented in the literature since 1980. While subcutaneous lipomas have been associated with hypercholesterolemia, obesity, and trauma, no such correlations have been established for lipomas that precipitate sciatic pain [10]. Typically, these tumors remain asymptomatic for prolonged periods before they attain substantial size, as observed in the present case that may cause displacement of pelvic organs or exert compressive effects on the sciatic nerve, leading to clinical symptoms. Diagnosis is primarily based on MRI or CT scans, though these modalities may not conclusively differentiate between a benign lipoma and a well-differentiated liposarcoma.

In this case, the deep lipoma was managed with complete surgical excision. Indications for excision include a mass > 5 cm, subfascial location, evidence of tumor growth, or clinical signs such as pain, firmness, or irregularity. Intramuscular lipomas, particularly those with an infiltrative growth pattern, demonstrate higher local recurrence rates, necessitating either total resection of the affected muscle or compartmental resection to reduce the risk of recurrence [11].

Sciatic hernia, first described by Papen in 1750, involves the herniation of abdominal contents through the greater or lesser sciatic foramen. Patients typically present with an uncomfortable mass in the gluteal region. A comprehensive literature review identified only 99 cases of sciatic hernia reported since 1900, underscoring its rarity as an etiology of sciatic neuralgia [12].

Various surgical approaches have been documented in the literature, including transgluteal, transabdominal, combined abdominal and gluteal, extraperitoneal, abdominoperineal, Kocher-Langenbeck, and laparoscopic techniques. Mehta N et al. approached leiomyosarcoma in the ischirectal fossa using combined transgluteal approach and trans-abdominal. This simultaneity allowed for a secure and comprehensive resection of the sarcoma, with preservation of critical anatomical structures. Moreover, this technique has the potential to significantly decrease operative duration [13]. For lesions affecting the proximal sciatic nerve near the sciatic notch, both the infragluteal and transgluteal approaches can be employed. In the study of Erhan Okay and Feyza Unlu Ozkan, when malignancy is suspected, the infragluteal approach is generally preferred over the transgluteal approach. This preference stems from the infragluteal approach's ability to provide extensive surgical margins while minimizing the risk of intercompartmental contamination, which is critical in oncologic resection [7]. In this case, surgical intervention was imperative for tumor removal. The transgluteal approach was selected, providing adequate visualization of the sciatic nerve and enabling the safe excision of the lipoma through blunt dissection performed with meticulous care [14].

However, a notable limitation of this approach is the partially blind dissection required for addressing the pelvic extension of the lipoma.

## Conclusion

The diverse range of neoplastic and non-neoplastic soft tissue masses within the retroperitoneal space mirrors the intricate anatomy of this region. Lipomas are encapsulated benign tumors arising from adipose tissue, which definitive treatment requires complete excision of the mass along with its capsule. Performing an excision via the transgluteal approach for a lipoma originating in the pelvic region and causing sciatic pain represents a novel and bold approach to treatment. This innovative approach underscores the importance of tailored surgical strategies in the management of complex retroperitoneal tumors.
